# Supplementary material for: Discovery and application of insertion-deletion (INDEL) polymorphisms for QTL mapping of early life-history traits in Atlantic salmon
Source: BMC Genomics. 2010 Mar 8;11:156. doi: 10.1186/1471-2164-11-156 (PMC2838853; doi:10.1186/1471-2164-11-156)
Supplement: Additional file 2 — Information on developed 76 locus single-run INDEL panel in Atlantic salmon. Information on fluorescence labeling, primer concentrations, PCR pooling and links to alignments, INDEL motifs and GENESCAN (Burge and Karlin 1997) predictions of genes/exons are available in html format. [file 1471-2164-11-156-S2.ZIP › Additionalfile2/table22471.html]

Contig Alignment

```
Base	A B C D   
1	A A . A    
2	C C . C    
3	T T . T    
4	G G . G    
5	A A . A    
6	C C . C    
7	A A . A    
8	T T . T    
9	G G . G    
10	G G . G    
11	G G . G    
12	T T . T    
13	T T . T    
14	A A . A    
15	A A . A    
16	T T . T    
17	T T . T    
18	G G . G    
19	A A . A    
20	G G . G    
21	T T . T    
22	G G . G    
23	A A . A    
24	C C . C    
25	T T . T    
26	G G . G    
27	A A . A    
28	C C . C    
29	C C . C    
30	T T . T    
31	A A . A    
32	A A . A    
33	C C . C    
34	G G . G    
35	A A . A    
36	G G . G    
37	A A . A    
38	G G . G    
39	A A . A    
40	A A . A    
41	A A . A    
42	C C . C    
43	C C . C    
44	G G . G    
45	C C . C    
46	T T . T    
47	G G . G    
48	A A . A    
49	T T . T    
50	G G . G    
51	C C . C    
52	A A . A    
53	G G . G    
54	T T . T    
55	T T . T    
56	A A . A    
57	T T . T    
58	A A . A    
59	T T . T    
60	A A . A    
61	T T . T    
62	T T . T    
63	T T . T    
64	C C . C    
65	G G . G    
66	A A . A    
67	A A . A    
68	A A . A    
69	T T . T    
70	G G . G    
71	A A . A    
72	C C . C    
73	A A . A    
74	T T . T    
75	C C . C    
76	T T . T    
77	T T . T    
78	G G . G    
79	T T . T    
80	A A . A    
81	T T . T    
82	A A . A    
83	T T . T    
84	T T . T    
85	C C . C    
86	T T . T    
87	T T . T    
88	T T . T    
89	A A . A    
90	A A . A    
91	C C . C    
92	T T . T    
93	C C . C    
94	A A . A    
95	A A . A    
96	C C . C    
97	A A . A    
98	A A . A    
99	T T . T    
100	A A . A    
101	A A . A    
102	G G . G    
103	T T . T    
104	T T . T    
105	G G . G    
106	A A . A    
107	G G . G    
108	A A . A    
109	C C . C    
110	C C . C    
111	C C . C    
112	C C . C    
113	A A . A    
114	G G . G    
115	C C . C    
116	G G . G    
117	G G . G    
118	A A . A    
119	C C . C    
120	A A . A    
121	T T . T    
122	T T . T    
123	T T . T    
124	C C . C    
125	T T . T    
126	G G . G    
127	A A . A    
128	G G . G    
129	T T . T    
130	T T . T    
131	T T . T    
132	C C . C    
133	C C . C    
134	G G . G    
135	G G . G    
136	C C . C    
137	A A . A    
138	T T . T    
139	G G . G    
140	A A . A    
141	C C . C    
142	G G . G    
143	T T . T    
144	G G . G    
145	A A . A    
146	A A . A    
147	T T . T    
148	A A . A    
149	T T . T    
150	A A . A    
151	T T . T    
152	A A . A    
153	T T . T    
154	G G . G    
155	A A . A    
156	A A . A    
157	G G . G    
158	A A . A    
159	C C . C    
160	A A . A    
161	T T . T    
162	T T . T    
163	G G . G    
164	T T . T    
165	A A . A    
166	A A . A    
167	T T . T    
168	C C . C    
169	T T . T    
170	A A . A    
171	T T . T    
172	T T . T    
173	T T . T    
174	A A . A    
175	A A . A    
176	T T . T    
177	A A . A    
178	T T . T    
179	A A . A    
180	A A . A    
181	T T . T    
182	A A . A    
183	A A . A    
184	T T . T    
185	G G . G    
186	T T . T    
187	G G . G    
188	C C . C    
189	A A . A    
190	T T . T    
191	T T . T    
192	C C . C    
193	T T . T    
194	G G G G    
195	A A A A    
196	G G G G    
197	T T T T    
198	C C C C    
199	T T T T    
200	T T T T    
201	T T T T    
202	T T T T    
203	C C C C    
204	G G G G    
205	G G G G    
206	T T T T    
207	C C C C    
208	A A A A    
209	G G G G    
210	C C C C    
211	C C C C    
212	A A A A    
213	G G G G    
214	A A A A    
215	T T T T    
216	G G G G    
217	T T T T    
218	T T T T    
219	T T T T    
220	T T T T    
221	A A A A    
222	G G G G    
223	C C C C    
224	T T T T    
225	C C C C    
226	G G G G    
227	A A A A    
228	T T T T    
229	T T T T    
230	C C C C    
231	A A A A    
232	C C C C    
233	T T T T    
234	G G G G    
235	A A A A    
236	A A A A    
237	A A A A    
238	A A A A    
239	G G G G    
240	A A A A    
241	G G G G    
242	A A A A    
243	C C C C    
244	G G G G    
245	T T T T    
246	T T T T    
247	C C C C    
248	A A A A    
249	T T T T    
250	T T T T    
251	T T T T    
252	G G G G    
253	A A A A    
254	C C C C    
255	T T T T    
256	C C C C    
257	C C C C    
258	C C C C    
259	A A A A    
260	A A A A    
261	A A A A    
262	C C C C    
263	G G G G    
264	G G G G    
265	C C C C    
266	T T T T    
267	C C C C    
268	T T T T    
269	T T T T    
270	T T T T    
271	T T T T    
272	G G G G    
273	T T T T    
274	T T T T    
275	C C C C    
276	A A A A    
277	G G G G    
278	T T T T    
279	G G G G    
280	G G G G    
281	T T T T    
282	G G G G    
283	C C C C    
284	T T T T    
285	T T T T    
286	A A A A    
287	A A A A    
288	A A A A    
289	A A A A    
290	A A A A    
291	T T T T    
292	G G G G    
293	T T T T    
294	A A A A    
295	T T T T    
296	G G G G    
297	A A A A    
298	G G G G    
299	A A A A    
300	A A A A    
301	A A A A    
302	T T T T    
303	C C C C    
304	A A A A    
305	A A A A    
306	A A A A    
307	T T T T    
308	A A A A    
309	T T T T    
310	T T T T    
311	T T T T    
312	C C C C    
313	T T T T    
314	A A A A    
315	A A A A    
316	T T T T    
317	G G G G    
318	T T T T    
319	A A A A    
320	A A A A    
321	C C C C    
322	G G G G    
323	C C C C    
324	C C C C    
325	T T T T    
326	A A A A    
327	A A A A    
328	A A A A    
329	G G G G    
330	G G G G    
331	G G G G    
332	T T T T    
333	T T T T    
334	G G G G    
335	C C C C    
336	C C C C    
337	T T T T    
338	A A A A    
339	C C C C    
340	C C C C    
341	A A A A    
342	T T T T    
343	T T T T    
344	A A A A    
345	T T T T    
346	G G G G    
347	T T T T    
348	C C C C    
349	A A A A    
350	G G G G    
351	A A A A    
352	T T T T    
353	T T T T    
354	G G G G    
355	T T T T    
356	G G G G    
357	A A A A    
358	C C C C    
359	A A A A    
360	T T T T    
361	G G G G    
362	T T T T    
363	G G G G    
364	A A A A    
365	G G G G    
366	T T T T    
367	T T T T    
368	C C C C    
369	A A A A    
370	A A A A    
371	A A A A    
372	T T T T    
373	A A A A    
374	T T T T    
375	A A A A    
376	A A A A    
377	A A A A    
378	A A A A    
379	A A A A    
380	A A A A    
381	- A - A  2	 indel  
382	T T T T    
383	T T T T    
384	C C C C    
385	T T T T    
386	G G G G    
387	A A A A    
388	C C C C    
389	G G G G    
390	A A A A    
391	A A A A    
392	A A A A    
393	T T T T    
394	T T T T    
395	A A A A    
396	A A A A    
397	A A A A    
398	T T T T    
399	T T T T    
400	G G G G    
401	T T T T    
402	G G G G    
403	T T T T    
404	G G G G    
405	C C C C    
406	A A A A    
407	A A A A    
408	A A A A    
409	A A A A    
410	A A A A    
411	A A A A    
412	A A A A    
413	- A - A  2	 indel  
414	G G G G    
415	A A A A    
416	C C C C    
417	T T T T    
418	T T T T    
419	T T T T    
420	G G G G    
421	A A A A    
422	A A A A    
423	T T T T    
424	G G G G    
425	A A A A    
426	G G G G    
427	G G G G    
428	C C C C    
429	T T T T    
430	T T T T    
431	T T T T    
432	C C C C    
433	T T T T    
434	C C C C    
435	C C C C    
436	T T T T    
437	C C C C    
438	A A A A    
439	C C C C    
440	T T T T    
441	A A A A    
442	A A A A    
443	C C C C    
444	T T T T    
445	A A A A    
446	T T T T    
447	G G G G    
448	T T T T    
449	T T T T    
450	T T T T    
451	T T T T    
452	T T T T    
453	A A A A    
454	T T T T    
455	A A A A    
456	A A A A    
457	C C C C    
458	T T T T    
459	T T T T    
460	A A A A    
461	T T T T    
462	C C C C    
463	T T T T    
464	G G G G    
465	C C C C    
466	A A A A    
467	G G G G    
468	A A A A    
469	A A A A    
470	T T T T    
471	G G G G    
472	T T T T    
473	C C C C    
474	G G G G    
475	T T T T    
476	T T T T    
477	G G G G    
478	T T T T    
479	C C C C    
480	T T T T    
481	G G G G    
482	G G G G    
483	A A A A    
484	G G G G    
485	C C C C    
486	T T T T    
487	C C C C    
488	A A A A    
489	G G G G    
490	A A A A    
491	A A A A    
492	A A A A    
493	A A A A    
494	G G G G    
495	T T T T    
496	A A A A    
497	G G G G    
498	C C C C    
499	A A A A    
500	G G G G    
501	T T T T    
502	A A A A    
503	A A A A    
504	C C C C    
505	T T T T    
506	G G G G    
507	T T T T    
508	C C C C    
509	T T T T    
510	G G G G    
511	C C C C    
512	A A A A    
513	A A A A    
514	A A A A    
515	T T T T    
516	C C C C    
517	A A A A    
518	G G G G    
519	A A A A    
520	G G G G    
521	G G G G    
522	C C C C    
523	A A A A    
524	G G G G    
525	C C C C    
526	C C C C    
527	A A A A    
528	A A A A    
529	A A A A    
530	T T T T    
531	G G G G    
532	A A A A    
533	A A A A    
534	C C C C    
535	G G G G    
536	T T T T    
537	A A A A    
538	T T T T    
539	C C C C    
540	T T T T    
541	T T T T    
542	T T T T    
543	C C C C    
544	A A A A    
545	C C C C    
546	A A A A    
547	G G G G    
548	A A A A    
549	C C C C    
550	G G G G    
551	C C C C    
552	A A A A    
553	A A A A    
554	T T T T    
555	T T T T    
556	T T T T    
557	G G G G    
558	A A A A    
559	T T T T    
560	T T T T    
561	C C C C    
562	C C C C    
563	C C C C    
564	G G G G    
565	A A A A    
566	C C C C    
567	G G G G    
568	T T T T    
569	T T T T    
570	C C C C    
571	A A A A    
572	C C C C    
573	C C C C    
574	A A A A    
575	A A A A    
576	A A A A    
577	A A A A    
578	A A A A    
579	G G G G    
580	A A A A    
581	G G G G    
582	C C C C    
583	C C C C    
584	G G G G    
585	T T T T    
586	T T T T    
587	C C C C    
588	G G G G    
589	T T T T    
590	T T T T    
591	C C C C    
592	T T T T    
593	G G G G    
594	G G G G    
595	T T T T    
596	C C C C    
597	C C C C    
598	G G G G    
599	A A A A    
600	C C C C    
601	C C C C    
602	A A A A    
603	C C C C    
604	T T T T    
605	A A A A    
606	T T T T    
607	T T T T    
608	A A A A    
609	T T T T    
610	A A A A    
611	T T T T    
612	- T - T  2	 indel  
613	- A - A  2	 indel  
614	- T - T  2	 indel  
615	- A - A  2	 indel  
616	- G - G  2	 indel  
617	A A A A    
618	A A A A    
619	A A A A    
620	G G G G    
621	T T T T    
622	T T T T    
623	A A A A    
624	T T T T    
625	C C C C    
626	G G G G    
627	G G G G    
628	C C C C    
629	A A A A    
630	A A A A    
631	G G G G    
632	C C C C    
633	A A A A    
634	G G G G    
635	C C C C    
636	G G G G    
637	A A A A    
638	G G G G    
639	C C C C    
640	A A A A    
641	C C C C    
642	T T T T    
643	A A A A    
644	T T T T    
645	A A A A    
646	A A A A    
647	A A A A    
648	T T T T    
649	G G G G    
650	G G G G    
651	T T T T    
652	A A A A    
653	G G G G    
654	T T T T    
655	T T T T    
656	A A A A    
657	A A A A    
658	G G G G    
659	G G G G    
660	T T T T    
661	A A A A    
662	T T T T    
663	A A A A    
664	A A A A    
665	T T T T    
666	T T T T    
667	T T T T    
668	A A A A    
669	G G G G    
670	G G G G    
671	G G G G    
672	G A A A  1	 transition  
673	. T . T    
674	. T . T    
675	. A . A    
676	. A . A    
677	. C . C    
678	. T . T    
679	. G . G    
680	. A . A    
681	. A . A    
682	. G . G    
683	. A . A    
684	. A . A    
685	. A . A    
686	. T . T    
687	. A . A    
688	. C . C    
689	. A . A    
690	. C . C    
691	. T . T    
692	. A . A    
693	. A . A    
694	. G . G    
695	. G . G    
696	. G . G    
697	. A . A    
698	. A . A    
699	. T . T    
700	. T . T    
701	. C . C    
702	. A . A    
703	. T . T    
704	. C . C    
705	. T . T    
706	. A . A    
707	. C . C    
708	. G . C  1	 transversion  
709	. . . T    
710	. . . G    
711	. . . C    
712	. . . C    
713	. . . C    
714	. . . A    
715	. . . G    
716	. . . T    
717	. . . G    
718	. . . T    
719	. . . A    
720	. . . G    
721	. . . T    
722	. . . C    
723	. . . C    
724	. . . C
```
